# Hypoxia induces protection against etoposide-induced apoptosis: molecular profiling of changes in gene expression and transcription factor activity

**DOI:** 10.1186/1476-4598-7-27

**Published:** 2008-03-26

**Authors:** Audrey Sermeus, Jean-Philippe Cosse, Marianne Crespin, Veronique Mainfroid, Francoise de Longueville, Noelle Ninane, Martine Raes, Jose Remacle, Carine Michiels

**Affiliations:** 1 URBC, FUNDP-University of Namur, 61 rue de Bruxelles, 5000 Namur, Belgium; 2 Eppendorf Array Technologies, 20A rue du Séminaire, 5000 Namur, Belgium

## Abstract

**Background:**

it is now well established that hypoxia renders tumor cells resistant to radio- but also chemotherapy. However, few elements are currently available as for the mechanisms underlying this protection.

**Results:**

in this study, physiological hypoxia was shown to inhibit apoptosis induced in HepG2 cells by etoposide. Indeed, hypoxia reduced DNA fragmentation, caspase activation and PARP cleavage. The DNA binding activity of 10 transcription factors was followed while the actual transcriptional activity was measured using specific reporter plasmids. Of note is the inhibition of the etoposide-induced activation of p53 under hypoxia. In parallel, data from low density DNA microarrays indicate that the expression of several pro- and anti-apoptotic genes was modified, among which are Bax and Bak whose expression profile paralleled p53 activity. Cluster analysis of data unravels several possible pathways involved in the hypoxia-induced protection against etoposide-induced apoptosis: one of them could be the inhibition of p53 activity under hypoxia since caspase 3 activity parallels Bax and Bak expression profile. Moreover, specific downregulation of HIF-1α by RNA interference significantly enhanced apoptosis under hypoxia possibly by preventing the hypoxia mediated decrease in Bak expression without altering Bax expression.

**Conclusion:**

these results are a clear demonstration that hypoxia has a direct protective effect on apoptotic cell death. Moreover, molecular profiling points to putative pathways responsible for tumor growth in challenging environmental conditions and cancer cell resistance to chemotherapeutic agents.

## Introduction

The adverse effect of tumor hypoxia on the outcome of clinical radiotherapy as well as chemotherapy is well established. Hypoxic conditions elicit cellular responses designed to improve cell survival through an adaptive process. Regulation of gene expression through HIF-1 (hypoxia-inducible factor-1) but also via other transcription factors plays an important role in this process. Moreover, these changes in gene expression enable tumors to take advantage of the physiological response mechanisms to hypoxia to improve their own survival as well as their metastatic properties [1].

HIF-1 is composed of two subunits belonging to the bHLH-PAS family: ARNT which is constitutively expressed in the nucleus and HIF-1α which is regulated by hypoxia. In normoxia, HIF-1α is hydroxylated on two prolines by oxygen-dependent prolyl hydroxylases and on one asparagine by an oxygen-dependent asparaginyl hydroxylase, FIH-1. The two hydroxylated prolines are recognized by the protein pVHL, which is part of an ubiquitin ligase complex, thus targeting the HIF-1α subunit for degradation by the proteasome. The hydroxylation on the asparagine prevents HIF-1α-CBP/p300 interaction. In low oxygen conditions, HIF-1α is no longer modified and is thus stabilized. HIF-1α then translocates into the nucleus where it dimerizes with ARNT. The products of HIF-1 target genes allow the cell to adapt to the hypoxic conditions [2,3].

Regions of hypoxia are evidenced within many solid tumors and the extent of tumor hypoxia is thought to be an important prognostic factor influencing tumor progression, resistance to therapy and overall patient survival [4-6]. A molecular explanation of these hypoxia-induced effects includes increased anaerobic glycolysis, induction of angiogenesis and increased expression of drug export pumps, e.g. MDR1 [7]. Many of these processes are regulated by HIF-1 [8].

If mild hypoxia is rather pro-survival, it must be noted however that severe or prolonged hypoxia can lead to cell death, mainly through an apoptotic pathway [9,10]. HIF-1 seems to play a major role in this process by inducing p53 stabilization [11,12], overexpression of pro-apoptotic proteins such as BNIP3 [13] or HGTD-P [14] as well as Bax translocation [15].

It is thus apparent that hypoxia can either initiate apoptosis and cell death or prevent cell death by provoking an adaptive response facilitating cell proliferation and tumor growth [16]. Considering that HIF-1 induces the expression of both pro-survival and cell death inducing genes, it is thus crucial to understand the fine tuning regulation that makes decision between life and death. Similarly, the influence of hypoxia on apoptosis resistance to radio- and chemotherapy still needs deeper understanding. The aim of this study was (i) to investigate the effect of hypoxia on the apoptosis induced by a drug used in chemotherapy, (ii) to define a molecular profiling of cancer cell response to this drug under normoxic and hypoxic conditions and (iii) to investigate the putative role of HIF-1 in these processes. Gene expression patterns were then correlated with the activity of several transcription factors including HIF-1 and p53 in order to define molecular pathway involved in the cellular response. We used etoposide as the apoptosis inducer. Etoposide is a topoisomerase II inhibitor that induces double strand breaks in DNA, thus leading to the activation of p53 and apoptosis [17].

## Results

### Hypoxia protects HepG2 cells against etoposide-induced apoptosis

**Figure 1:**
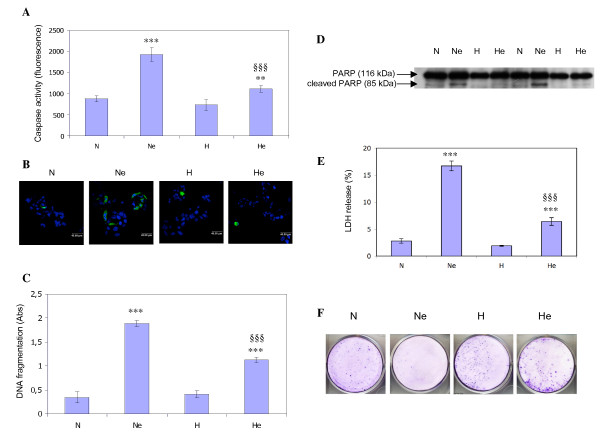
Hypoxia protects HepG2 cells against etoposide-induced apoptosis. HepG2 cells were incubated under normoxic (N) or hypoxic (H) conditions with or without etoposide (e) at 50 μM for 16 hours (A, B, C, D, F) or 40 hours (E). *A*, the overall caspase activity was assayed by measuring free rhodamine 110 released after cleavage of the caspase substrate DEVD-R110. Results are expressed as means ± 1 SD (n = 3). *B*, after the incubation, cells were fixed, permeabilized and stained for active caspase 3 using a specific antibody (green). Nuclei were detected with To-Pro-3 (blue). Observation was performed using a confocal microscope with a constant photomultiplier. *C*, after the incubation, DNA fragmentation was assayed using an ELISA for soluble nucleosomes. Results are expressed as means ± 1 SD (n = 3). *D*, PARP-1 and cleaved 85 kDa fragment were detected in total cell extracts by western blotting, using a specific mouse anti-PARP-1 antibody. *E*, after the incubation, LDH was assessed. Results are expressed as means ± 1 SD (n = 3). *F*, after the incubation, complete medium was added back and cells were incubated for 7 days before being fixed and stained with crystal violet. **, ***: p < 0.01, p < 0.001 vs. normoxia ; §§§: p < 0.001 vs. normoxia+etoposide.

Etoposide is known to induce apoptosis through DNA damage induced p53 activation [17]. HepG2 cells incubated in the presence of 50 μM etoposide during 16 hours did indeed undergo apoptosis as shown by an increase in caspase activity, in active caspase 3 abundance, in PARP cleavage and in DNA fragmentation (Fig. 1). Hypoxia alone did not induce apoptosis since no increase in any of these parameters was observed after 16 hours incubation. On the other hand, hypoxia inhibited the etoposide-induced apoptosis: a marked decrease in caspase activity and DNA fragmentation was observed in addition to a nearly complete inhibition of PARP cleavage and caspase 3 activation (Fig. 1). Cell death was also investigated after a longer period of incubation in order to investigate whether the effect of hypoxia was sustained. Cells were incubated in the presence of etoposide under normoxia or hypoxia for 40 hours and LDH release was assessed. An increase in cell mortality was observed in the presence of etoposide which was significantly inhibited by hypoxia (Fig. 1E). Clonogenic assays also revealed that the hypoxia-induced protection from apoptosis was translated into actual survival of the cells (Fig. 1F).

### Hypoxia and etoposide induce changes in gene expression

The apoptosis induced by etoposide is controlled by numerous pro- and anti-apoptotic genes. We hypothesized that hypoxia could induce gene expression alterations that would then inhibit the etoposide-induced apoptosis. We thus compared differences and similarities in gene expression modifications in both conditions i.e. etoposide under normoxic and hypoxic conditions, using a low-density DNA microarray that comprises 123 capture probes allowing gene expression analysis for a set of key genes related to apoptosis (DualChip^® ^human apoptosis, Eppendorf). HepG2 cells were incubated during 16 hours in the presence or in the absence of etoposide at 50 μM under normoxia or hypoxia. Three independent experiments were performed for each condition and each sample was hybridized to three submicroarrays represented by the probes spotted in triplicate.

Gene expression data are presented in Table 1. The first list identified 35 genes found to be significantly up-regulated for at least one experimental condition. Some of the genes upregulated by etoposide, are known p53 target genes (*GADD45, BAX, MDM2*) while some of the genes upregulated by hypoxia alone are known HIF-1 target genes (*BNIP3, ALDOA*). The increased expression of the genes induced by hypoxia did not seem to be affected by the presence of etoposide. These results are coherent with the fact that etoposide did not influence HIF-1 activity under hypoxia (see below). The upregulation of gene expression induced by etoposide was (*BAX, MDM2, BCL2A1*) or was not (*TNFRSF6*) affected by hypoxia, indicating that hypoxia may influence some but not all the etoposide-activated pathways. There are also genes upregulated both by etoposide and by hypoxia alone (*MCL1, BIRC3, IGFBP4*) and the combined effect of both conditions may be additive for some but not all of them (*CLK1, MCL1*). There are also 23 genes that were not really affected by etoposide but were down-regulated by hypoxia (second list). The presence of etoposide did not influence the hypoxia-induced down-regulation of these genes. Finally, the third list shows 7 genes that were upregulated by etoposide, down-regulated by hypoxia (except *AKT2 *and *E2F5*) and displayed a different behavior when both etoposide and hypoxia were applied. *BAK1 *is interesting since it is a pro-apoptotic gene belonging to the Bcl-2 family which is up-regulated when apoptosis is induced (in the presence of etoposide) and down-regulated under hypoxia, when apoptosis was decreased.

**Table 1:** Gene expression profiling in HepG2 cells incubated with or without etoposide under normoxic or hypoxic conditions.

	**N**	**Ne**	**H**	**He**
**BIRC3**	1	**2.99**	**1.53**	**2.31**
**CLK1**	1	**2.77**	**2.28**	**4.03**
**GADD45A**	1	**4.32**	**1.47**	**3.54**
**IGF2**	1	**2.67**	**1.59**	**1.57**
**IGFBP4**	1	**4.35**	**1.77**	**2.39**
**MCL1**	1	**3.44**	**3.92**	**5.27**
**ALDOA**	1	1.00	**1.43**	**1.59**
**BAG1**	1	2.07	**1.37**	**2.16**
**BIRC2**	1	2.42	**1.51**	**2.36**
**BIRC4**	1	1.25	**1.69**	**1.43**
**BNIP3**	1	1.19	**3.97**	**4.25**
**ING**	1	1.48	**1.83**	**1.97**
**JUN**	1	1.66	**2.33**	**3.28**
**MAP2K1**	1	1.38	**2.16**	**2.05**
**TANK**	1	1.77	**1.34**	**1.60**
**BAX**	1	**2.45**	0.88	**1.44**
**IGFBP2**	1	**1.83**	1.21	**1.30**
**MDM2**	1	**4.75**	1.44	**2.47**
**TNFRSF6**	1	**5.70**	0.69	**5.11**
**CASP1**	1	2.84	1.00	**1.71**
**GAPD**	1	1.13	1.43	**1.68**
**K-ALPHA-1**	1	1.03	0.78	**0.73**
**RPS9**	1	1.30	1.19	**1.48**
**CDK7**	1	1.60	**1.60**	1.52
**NFKB1**	1	1.27	**1.40**	1.25
**BCL2A1**	1	**2.01**	0.85	1.27
**CDKN1C**	1	**1.45**	1.06	0.90
**CDKN2B**	1	**1.64**	0.98	0.81
**MAPK12**	1	**1.96**	1.10	1.06
**RBL2**	1	**1.88**	1.03	0.99
**RBP1**	1	**2.05**	1.01	1.16
**RIPK1**	1	**1.58**	0.87	1.00
**TP53**	1	**1.82**	0.68	0.85
**TP73**	1	**1.62**	1.06	0.94
**YWHAZ**	1	**2.62**	0.82	0.81
**BIRC5**	1	1.07	0.68	0.66
**CDC25C**	1	2.17	0.60	0.59
**CDK2**	1	1.14	0.73	0.76
**MAPK9**	1	1.15	0.76	0.67
**TFDP1**	1	1.23	0.69	0.63
**GSR**	1	0.72	0.92	0.60
**GSA**	1	0.51	0.67	0.69
**BID**	1	1.50	0.83	1.06
**BIK**	1	2.09	0.63	0.92
**CDK9**	1	1.48	0.75	0.72
**CLU**	1	1.54	0.81	1.09
**BclX**	1	1.85	1.11	0.81
**CASP2**	1	1.12	0.83	0.81
**CDK4**	1	0.72	0.98	0.74
**CDK5R1**	1	1.43	0.87	0.75
**CDK6**	1	1.18	1.30	0.52
**CSE1L**	1	1.05	1.01	0.71
**GRB2**	1	1.31	0.83	0.72
**IGF1R**	1	1.26	1.10	0.66
**LTB**	1	1.38	0.97	0.82
**PLK**	1	1.14	0.81	0.46
**TNFSF7**	1	1.50	1.00	0.74
**TRAF3**	1	1.40	0.89	0.76
**BAK 1**	1	**3.42**	0.75	**2.13**
**CDKN1A**	1	**3.02**	0.52	**1.64**
**GPX1**	1	**2.88**	0.69	**1.43**
**CDC2**	1	**3.22**	0.74	0.83
**PCNA**	1	2.23	0.62	**1.68**
**AKT2**	1	**1.55**	1.00	0.78
**E2F5**	1	1.20	**1.90**	0.61

Cells were incubated under normoxic (N) or hypoxic (H) conditions with or without etoposide (e, 50 μM) for 16 hours before RNA extraction, reverse-transcription and cDNA hybridization, as described in Materials and Methods. Each value is the average of three ratio values calculated from three independent experiments. Mean ratios indicate a fold-increase or decrease in gene expression.

**Value **higher than 1 + 1 SD, Value lower than 1 - 1 SD

The fact that our data are in good agreement with previous studies reporting modifications in HIF-1 target gene expression during hypoxia as well as in p53 target genes in the presence of etoposide already validates the DNA microarray used in this study. In order to further validate our data, we also performed SYBR Green quantitative real time PCR assays for some selected genes. Values obtained for a set of 5 genes that were up-regulated or down-regulated in response to at least one condition were confirmed (Table 2). For these genes, we found a very good correlation between relative transcript abundance data obtained by DNA microarray and by real time PCR.

**Table 2:** Comparison of the results obtained with real time RT-PCR and DNA microarrays analyses for *MCL1, BAK1, GPX1, CDNK1A *and *JUN *genes.

		**N**	**Ne**	**H**	**He**
**MCL1**	real time RT-PCR	1	2.97	5.53	8.24
	DNA microarray	1	3.44	3.92	5.27
**BAK 1**	real time RT-PCR	1	3.37	0.96	2.92
	DNA microarray	1	3.42	0.75	2.13
**GPX1**	real time RT-PCR	1	4.04	0.94	2.21
	DNA microarray	1	2.88	0.69	1.43
**CDKN1A**	real time RT-PCR	1	3.65	0.72	2.44
	DNA microarray	1	3.02	0.52	1.64
**JUN**	real time RT-PCR	1	1.45	5.54	4.36
	DNA microarray	1	1.66	2.33	3.28

Cells were incubated under normoxic (N) or hypoxic (H) conditions with or without etoposide (e, 50 μM) for 16 hours. After the incubation, total RNA was extracted, submitted to reverse transcription and then to hybridization on the DNA microarrays or to amplification in the presence of SYBR Green and specific primers. a-tubulin was used as the house keeping gene for data normalization. Data are given in fold-induction.

### Hypoxia and etoposide induce changes in the activity of several transcription factors

The transcription of some of the etoposide-induced genes is regulated by p53 but there are also changes in gene expression observed in p53-negative cells [18]. Moreover, other transcription factors than HIF-1 are activated under hypoxic conditions [19]. We also observed changes in the expression of genes for which it is not yet known which transcription factor is involved. We thus investigated the activity of 10 transcription factors using several approaches to follow their nuclear abundance, their DNA binding activity and their transcriptional activity using reporter systems.

Immunofluorescence staining and confocal microscopy studies were performed to determine the abundance and the subcellular localization of the most relevant subunits of these transcription factors. The results show that HIF-1α was only present under hypoxic conditions. It was located exclusively in the nuclei and etoposide did not interfere with HIF-1α stabilization under hypoxia [additional file 1]. The expression of p53 was similarly investigated: p53 is present in the cytosol of unstimulated cells, etoposide markedly increased its abundance and induced its translocation into the nucleus. On the other hand, hypoxia decreased its abundance and in these conditions, etoposide also increased its abundance and induced its translocation into the nucleus, but to a lesser extent than in normoxia [additional file 1]. Upon DNA damage, p53 is activated by phosphorylation, serine 15 being the most important residue being modified [20]. Immunofluorescence staining for the serine 15 phosphorylated form of p53 showed that this form of p53 was not present in unstimulated cells, was slightly induced in the nucleus of hypoxic cells and highly increased in the nucleus of cells incubated in the presence of etoposide under both normoxia and hypoxia [additional file 1]. These results indicate that the level of phosphorylation of p53 on serine 15 did not follow the abundance of the protein: indeed this level is enhanced under hypoxia while the protein abundance was decreased. A third transcription factor involved in transcriptional response to hypoxia [21] as well as to DNA damage [22] is AP-1. One of the most frequent subunit is c-jun and c-jun needs to be phosphorylated in order to be active. Hypoxia and to a larger extent etoposide, both increased c-jun protein abundance. There was no additive effect of both conditions and the protein was located in the nucleus [additional file 1]. On the other hand, etoposide induced c-jun phosphorylation on serine 63 while hypoxia had no effect and did not influence the effect of etoposide. As for p53, there was no correlation between c-jun protein abundance and its level of phosphorylation. Hypoxia also increased the abundance of MEF-2 while it decreased phospho-CREB and c-myc abundance and had no effect on phospho-elk1, phospho-ATF2, STAT-1. It had also different effects on the abundance of the different subunits constituting the NF-kB factor [additional file 1] (Table 3).

**Table 3:** The table summarizes the effects of hypoxia and/or etoposide on the abundance and the activity of 10 transcription factors.

			**N**	**Ne**	**H**	**He**
**p53**		TFChipMAPK	++	+++	+	++
		Trans-AM	++	+++	+	++
		prot level	++ (cyt)	+++ (cyt+nuc)	+ (cyt)	++ (cyt+nuc)
	P-p53	prot level (nuc)	0	+++	+	+++
		reporter system	++	+++	+	++
**AP-1**	c-jun	Trans-AM	+	+	++	+++
		prot level (nuc)	+	+++	++	+++
	P-c-jun	TFChipMAPK	+	+	++	+++
		prot level (nuc)	+	++	+	++
	c-fos	Trans-AM	+	+++	++	+++
		prot level (cyt+nuc)	+	+++	++	+++
		reporter system	+	+	++	+++
**P-Elk-1**		TFChipMAPK	++	++	+	++
		Trans-AM	++	++	+	+
		prot level (nuc)	+	+	+	+
**CREB/ATF2**	P-ATF-2	TFChipMAPK	++	+	++	++
		prot level (cyt+nuc)	+	+	+	++
	P-CREB	Trans-AM	+	+	+	+
		prot level (cyt+nuc)	++	++	+	+
		reporter system	+	+	+++	+
**MEF-2**		TFChipMAPK	++	++	+	+
		prot level	+ (cyt+nuc)	+++ (nuc)	++ (cyt+nuc)	+ (nuc)
**c-myc**		TFChipMAPK	++	0	+	0
		Trans-AM	+++	++	+	0
		prot level (nuc)	+++	+	++	+
		reporter system	++	0	+	0
**STAT-1**		TFChipMAPK	0	0	0	+
		prot level	+ (cyt)	+ (cyt)	+ (cyt)	+++ (cyt+nuc)
**NFATc1**		TFChipMAPK	0	0	0	0
**NF-kB**	c-rel	prot level (cyt+nuc)	++	++	++	++
	p50	prot level (cyt+nuc)	+++	++	+	++
	p65	Trans-AM	++	+++	+	+
		prot level (cyt)	+	+	++	+
		reporter system	+	++	+	++
**HIF-1**	HIF-1α	prot level (nuc)	0	0	++	++
		Trans-AM	+	+	+++	+++
		reporter system	+	+	+++	+++

Cyt: cytosolic, nuc: nuclear, 0: not present or no activity.

Transcription factor activity is regulated not only at the level of their expression but also by post-translational modifications as well as by protein-protein interactions. In a first approach to study the activity of the transcription factors of interest, we measured their DNA binding activity in vitro. To investigate the DNA binding activity of the transcription factors that are differentially regulated in these conditions, a multiplex assay has been used. This microarray comprises double-stranded DNA molecules fixed in triplicate on the support. Two types of DNA molecules are spotted: (i) "wild-type" DNA carrying the specific recognition sequence for the respective transcription factor and (ii) "mutated" DNA carrying point mutations that will not be recognized by the factor. Probes allowing the assay of the DNA binding activity for 8 transcription factors are present on the array (TFChip MAPK kit, Eppendorf). HepG2 cells were incubated 16 hours in the presence or in the absence of etoposide at 50 μM under normoxia or hypoxia. Nuclear extracts were recovered and hybridized on the microarray. Detection is performed by a cocktail of nine primary antibodies, one for each activated transcription factor and one recognizing the positive detection control, and labeled secondary antibodies. Three independent experiments were performed for each condition.

**Figure 2:**
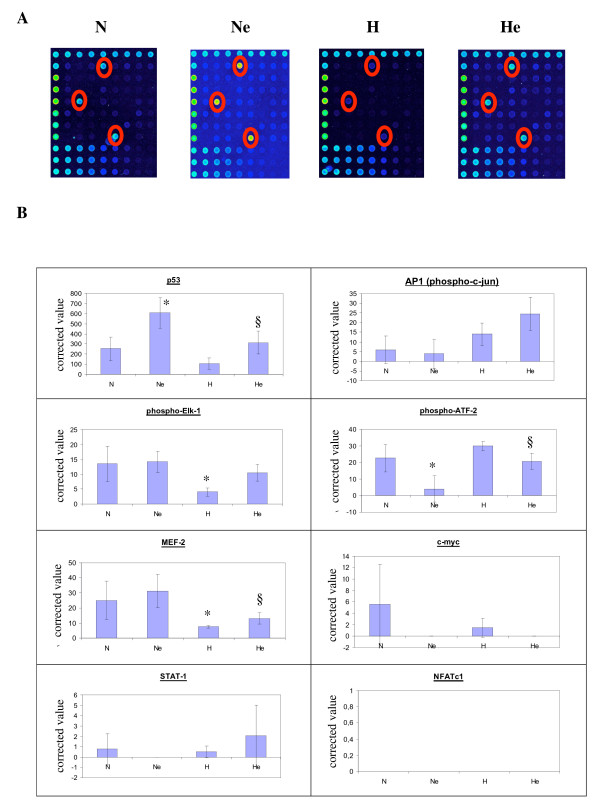
Effect of hypoxia and/or etoposide on DNA binding activity of 8 transcription factors, measured with the TF Chip MAPK microarrays. Cells were incubated under normoxic (N) or hypoxic (H) conditions with or without etoposide (e) at 50 μM for 16 hours. After the incubation, nuclear extracts were performed and hybridized on the arrays as described in Materials and Methods. *A*, Arrays hybridized with nuclear extracts from each condition. Fluorescence is represented in pseudocolor scale and corresponds to the DNA binding activity. Red circles point out spots for detecting p53 DNA binding. *B*, each value is the average of the corrected values calculated from three independent experiments and expressed as mean ± 1 SD (n = 3). *, **, ***: p < 0.05, p < 0.01, p < 0.001 vs. normoxia ; §: p < 0.05 vs. normoxia+etoposide.

An example of such an experiment is presented in Fig. 2A. p53 was the most abundant of the 8 transcription factors. The three spots corresponding to the binding of p53 to its wild-type DNA probe are clearly visible on each array, as well as the difference in fluorescence intensity according to the experimental conditions. Figure 2B shows the quantitative data. There was no or very low DNA binding for c-myc, STAT-1 and NFATc1. DNA binding activity of elk-1 and MEF-2 decreased under hypoxic conditions while etoposide had no effect. Etoposide seemed to decrease ATF-2 DNA binding activity. Interesting data were obtained for AP-1 and p53: hypoxia increased AP-1 DNA binding activity, which was further enhanced in the presence of etoposide while etoposide had no effect under normoxic conditions. On the other hand, etoposide markedly increased p53 DNA binding activity while hypoxia inhibited it. An intermediate value was obtained in the presence of etoposide under hypoxic conditions.

In order to confirm these data, individual DNA binding assays (TransAM assay) were performed for some of these factors and for others which were not detectable with the array [additional file 2]. Similar profiles of DNA binding activity than the ones obtained with the array were observed for c-myc, elk-1 as well as for p53 and AP-1, using an antibody directed against c-jun. CREB DNA binding activity was not changed by any treatment. NF-kB DNA binding activity was diminished by hypoxia but enhanced by etoposide while HIF-1 activity was greatly enhanced by hypoxia with no influence of etoposide.

**Figure 3:**
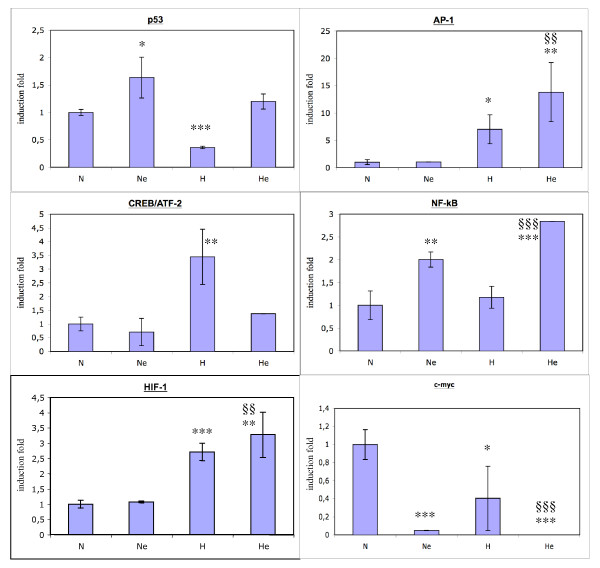
Effect of hypoxia and/or etoposide on the transcription activity of 6 transcription factors, measured with a reporter assay. Cells were cotransfected with the corresponding reporter plasmid encoding the firefly luciferase and the pCMVβ normalization plasmid before being incubated 16 hours under normoxia (N) or hypoxia (H) in the presence or absence of etoposide (e) at 50 μM. Results are expressed as means of the ratio between firefly luciferase activity and the β-galactosidase activity ± 1 SD (n = 3). Results are expressed in induction levels by comparison with the reference condition, normoxia. *, **, *** : p < 0.05, p < 0.01, p < 0.001 vs. normoxia ; §§, §§§: p < 0.01, p < 0.001 vs. normoxia+etoposide.

Finally, actual transcriptional activity was measured using a specific reporter system for 6 transcription factors (Fig. 3): transcriptional activity of p53, AP-1, HIF-1 and c-myc was parallel to their DNA binding activity assayed by the array and/or the TransAM assay. CREB and ATF-2 recognize the same DNA sequence and the results obtained with a reporter system containing this sequence shows an increase in luciferase expression under hypoxic conditions. Since there was no change in phospho-CREB and phospho-ATF-2 DNA binding activity nor in their nuclear abundance, this increase was probably due to the activation of another member of this family yet to be identified. Discrepancies were also observed for NF-kB since its transcriptional activity did not fit the DNA binding activity of p65. Here also another subunit may be involved. All these results indicate that hypoxia and etoposide have profound and different effects, alone and in combination, on the activity of numerous transcription factors. Table 3 summarizes all these data.

### Cluster analysis

**Figure 4:**
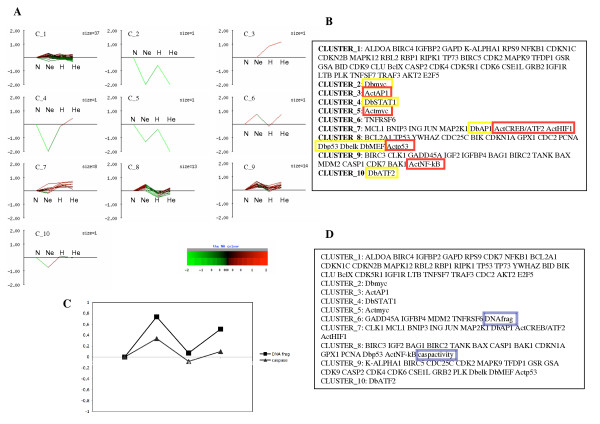
Cluster analysis of the gene expression patterns and transcription factor activity profiles. The expression values of up- and down-regulated genes as well as the activity profiles of the transcription factors of interest, measured with the DNA binding array and using a reporter system, were subjected to K-means clustering generating 10 clusters. *A*. Pseudocolors are given for increased expression/activity (red) or decreased expression/activity (green). *B*, Act– (highlighted in red) means the transcriptional activity measured by a reporter system for each transcription factor of interest; Db– (highlighted in yellow) is for the DNA binding activity measured with the array. *C, D *Cluster analysis of the gene expression patterns, the transcription factor activity profiles and the "phenotypic" profiles of the apoptosis level. *C*, "phenotype" values from figure 1, for caspase activity (caspactivity) and DNA fragmentation (DNAfrag) (highlighted in blue), were transformed in log values of the ratio normalized to the corresponding normoxic control. *D*, these values were subjected to K-means clustering generating 10 clusters. Act– means the transcriptional activity measured by a reporter system for each transcription factor of interest; Db– is for the DNA binding activity measured with the array.

Gene expression microarrays generate a large sum of data. In addition, we also assessed the activity of 10 transcription factors in the four different experimental conditions studied here. In order to investigate which transcription factor would be responsible for the changes in expression observed for the different genes, cluster analysis was performed. The aim was to search for similar variation patterns. The expression values of up- and down-regulated genes as well as the activity profiles of the transcription factors of interest, measured with the DNA binding array and using a reporter system, were subjected to K-means clustering generating 10 clusters. These 10 clusters are illustrated in Fig. 4A and 4B. DNA binding activity and transcriptional activity were put in the same cluster for p53 but not for AP-1 and c-myc despite very similar profiles. This is due to the transformation of the actual values in log generating negative values if the original one is lower than the corresponding normoxic control. ATF-2 DNA binding activity was not either clustered with ATF-2/CREB transcriptional activity, probably because, as mentioned earlier, another subunit of this family was involved to increase luciferase activity under hypoxia. An interesting result was obtained for cluster 7: the expression profile of five genes were clustered with the activity of HIF-1. Three of these genes, *MCL1, BNIP3 *and *JUN*, are known to be HIF-1-target genes (respectively [23,13,24]), thus validating this bioinformatics approach. Similarly, cluster 8 correlated the activity of p53 with the expression of several of its target genes: *CDKN1A, GPX1 *and *PCNA*, as well as its own encoding gene (*TP53*). It has to be mentioned that the expression profile of other p53 target genes were clustered in another cluster (cluster 9): *BAX, MDM2 *and *GADD45A*. Cluster 8 contains genes whose expression was significantly diminished under hypoxia while the expression of those of cluster 9 was either unchanged or increased in these conditions. NF-kB activity was also clustering in the latter, together with IAP encoding genes (*BIRC2 *for cIPA-1 and *BIRC3 *for cIAP-2), whose expression is known to be regulated by this transcription factor [25,26].

The ultimate goal of this study is to understand how hypoxia could decrease etoposide-induced apoptosis. In order to have a first insight in these hypoxia-induced mechanisms, we performed a second cluster analysis incorporating "phenotype" values from figure 1 (caspase activity and DNA fragmentation) expressed in log values of the ratio normalized to the corresponding normoxic control (Fig. 4C). Fig. 4D shows a summary of the 10 clusters. Clusters 8 and 9 contain p53 activity data, expression profile of most p53 target genes as well as data for caspase 3 activity. On the other hand, data for DNA fragmentation clustered with the expression profile of *GADD45A*, *MDM2 *and *TNFRSF6 *(Fas), three p53 target genes. There was no correlation of these phenotypic profiles with AP-1 or HIF-1 activity.

### Effect of HIF-1α silencing on the hypoxia-induced protection

**Figure 5:**
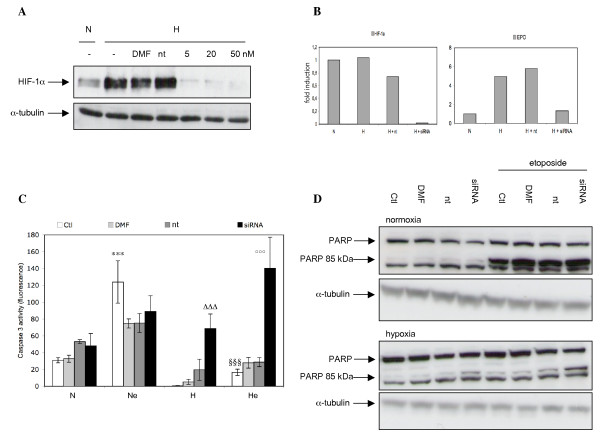
Effect of HIF-1α silencing on the hypoxia-induced protection against the etoposide-induced apoptosis. *A*, cells were transfected with 5, 20 or 50 nM HIF-1α siRNA, 50 nM non-targeting siRNA or with the transfection reagent alone (DMF) for 24 hours. Cells were then incubated under normoxia or hypoxia for 6 hours and total cell extracts were analyzed by western blot for HIF-1α protein level. *A, B, C*, cells were transfected with 50 nM HIF-1α siRNA or non-targeting siRNA for 24 hours. They were then incubated under normoxic (N) or hypoxic (H) conditions with or without etoposide (e, 50 μM) for 16 hours. *B*, after the incubation, total RNA was extracted, submitted to reverse transcription and then to amplification in the presence of SYBR Green and specific primers. α-tubulin was used as the house keeping gene for data normalization. Data are given in fold-induction. *C*, caspase 3 activity was assayed. Results are expressed as means ± 1 SD (n = 3). *** p < 0.001 vs. normoxia ; §§§ p < 0.001 vs. normoxia+etoposide ; ΔΔΔ p < 0.001 vs. hypoxia ; °°° p < 0.001 vs. hypoxia+etoposide. *D*, PARP-1 and cleaved 85 kDa fragment were detected in total cell extracts by western blotting, using a specific mouse anti-PARP-1 antibody.

HIF-1 and p53 interact in many ways to finally dictate cell fate. We investigated whether HIF-1 was involved in the hypoxia-induced protection against the etoposide-induced apoptosis. For that, HIF-1α expression was silenced through RNA interference. Fig. 5A and 5B show that 50 nM HIF-1α siRNA inhibited HIF-1α protein expression and decreased mRNA level by more than 95 %. This was translated into an inhibition of HIF-1 target gene overexpression under hypoxic conditions as shown for EPOmRNA level (Fig. 5B). HIF-1α siRNA completely prevented the hypoxia-induced protection against the etoposide-induced apoptosis as measured by caspase 3 activity (Fig. 5C) or PARP cleavage (Fig. 5D): the protection was completely lost when HIF-1α expression was inhibited. The non-targeting siRNA did not influence apoptosis. It has to be noted that HIF-1α siRNA also increased cell death under hypoxia alone (without etoposide), which is understandable knowing that HIF-1α is the main regulator of cell adaptation to hypoxia. All together, these results indicate that HIF-1α is necessary for the hypoxia-induced resistance to apoptosis.

**Figure 6:**
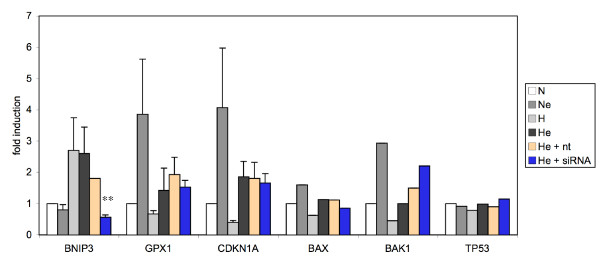
*A*, Gene expression profiling in HepG2 cells incubated with or without etoposide under normoxic or hypoxic conditions after HIF-1α siRNA transfection. Cells were transfected with 50 nM HIF-1α siRNA or non-targeting siRNA for 24 hours. They were then incubated under normoxic (N) or hypoxic (H) conditions with or without etoposide (e, 50 μM) for 16 hours before total RNA extraction, reverse transcription and amplification by real-time PCR in the presence of SYBR Green and specific primers. α-tubulin was used as the house keeping gene for data normalization. Data are given in fold-induction as the mean ± 1 SD for experimental triplicates or as the mean for experimental duplicates. ** p < 0.01 vs hypoxia+etoposide.

To gain further insight into modulation of apoptosis pathways by HIF-1α, we next assessed the effect of knockdown of HIF-1α on mRNA levels of pro- and anti-apoptotic proteins, using the low density DNA microarray used hereabove. HIF-1α siRNA completely prevented the upregulation of *BNIP3 *mRNA level induced by etoposide and hypoxia. This observation validated the effect of the siRNA since *BNIP3 *is known to be a HIF-1 target gene. Interestingly, we observed that HIF-1α siRNA prevented the hypoxia-induced decrease in *BAK1 *mRNA level without affecting *BAX *expression (Fig. 6A). It has to be noted that HIF-1α siRNA had no influence on *TP53 *mRNA level. All these results were confirmed by real-time RT-PCR assays (Table 4).

**Table 4:** Gene expression profiling in HepG2 cells incubated with or without etoposide under normoxic or hypoxic conditions after HIF-1α siRNA transfection.

	**N**	**Ne**	**He**	**He + siRNA**
**CLK1**	1	5.30	19.70	6.57
**CGADD45A**	1	1.33	0.71	1.01
**IGFPB4**	1	**2.06**	1.21	**2.14**
**MCL1**	1	**1.69**	1.16	**2.09**
**BIRC2**	1	0.73	0.41	0.44
**BIRC4**	1	0.76	0.35	0.25
**BIRC3**	1	0.93	**2.52**	0.35*
**ING**	1	1.08	0.41	0.43
**JUN**	1	1.50	1.23	2.66
**MAP2K1**	1	0.82	0.58	0.78
**TANK**	1	5.42	0.38	0.37
**BAX**	1	**1.53**	0.59	0.87
**IGFBP2**	1	1.13	0.77	0.92
**MDM2**	1	**3.90**	1.28	2.28
				
**BAK1**	1	**2.35**	0.69	1.69*
**CDKN1A**	1	**2.61**	0.65	1.41
**GPX1**	1	**2.27**	0.59	1.09
**CDC2**	1	0.89	0.29	0.35
**PCNA**	1	**2.11**	0.49	1.06
**AKT2**	1	**4.46**	2.31	1.90
**E2F5**	1	1.23	0.24	0.14

Cells were transfected with 50 nM HIF-1α siRNA or non-targeting siRNA for 24 hours. They were then incubated under normoxic (N) or hypoxic (H) conditions with or without etoposide (e, 50 μM) for 16 hours before RNA extraction, reverse-transcription and cDNA hybridization, as described in Materials and Methods. Each value is the average of three ratio values calculated from three independent experiments. Mean ratios indicate a fold-increase or decrease in gene expression.

**Value **higher than 1 + 1 SD, Value lower than 1 – 1 SD, * p < 0.05 vs He

## Discussion

A major drawback of the treatment of cancer patients with chemotherapeutic agents is the development of resistance. Besides the overexpression of ABC transporters and the selection of mutated cells whose apoptotic process is deficient, hypoxia has been shown to impair the effect of these molecules [16,27,28]. Indeed, while severe and prolonged hypoxia may initiate apoptosis, cells often adapt to mild hypoxia and survive. Hypoxia by itself can also prevent apoptosis induced by several agents such as the ones used in chemotherapy [9,10]. However, the exact mechanisms responsible for this protective effect of hypoxia still remain elusive.

Using a simple in vitro experimental model with hepatoma HepG2 cells as an example of cancer cells and etoposide, a chemotherapeutic agent used in clinic, we demonstrated that etoposide did indeed induced apoptosis in HepG2 cells and that hypoxia effectively prevents cell death (Fig. 1, [29]). Similar protective effects of hypoxia have also been reported for oxidative stress-induced apoptosis in HepG2 cells [23,30], for cisplatin and doxorubicin-induced cell death in non-small cell lung cancer [31] and for cisplatin-induced apoptosis in tubular cells [32].

In this study, we aimed to define mechanisms initiated by hypoxia which are responsible for its protective effects. Gene expression data evidenced several pro- and anti-apoptotic genes whose expression was upregulated by etoposide with the expression of some of them being further modulated by hypoxia. As a DNA damaging reagent, etoposide has been shown to induce apoptosis in a variety of cell lines harboring either wildtype, like HepG2 cells, or mutated p53 [33,34]. Although the entire signaling pathway is not yet known, one clearly involves p53 [35]. Our data indeed evidenced upregulation of p53 target genes such as *GADD45, BAX, MDM2, CDKN1A *(p21), *GPX1 *and *TNFRSF6 *(Fas). The expression of these genes was also shown to be enhanced after etoposide treatment in U2-OS cells, that also contain wildtype p53, in another DNA microarray study [36]. Another cDNA array analysis performed in p53 mutated cells, HL60, identified 40 genes differentially expressed by etoposide including *MCL1 *and *JUN *[18]. The expression of these genes is thus clearly p53 independent in this case. Their expression has also been observed to increase in our study.

Hypoxia alone also strongly influenced gene expression. Besides known HIF-1 target genes like *BNIP3 *[13], *MCL1 *[23], *JUN *[24] or *aldolase *[37], other genes were also upregulated, such as CLK1 encoding a kinase, *IGFBP4 *and *BIRC2*, *3 *and *4 *encoding different inhibitors of apoptosis. Moreover, hypoxia also down- or up-regulated the expression of some of the etoposide responsive genes respectively *IGFBP4, BAX, BAK1, MDM2, CDKN1A, GPX *and *CLK1*, *MCL1*. On the other hand, it had no effect on the expression of others like *TNFRSF6, GADD45A *and *BIRC3*. We conclude from these observations that one putative mechanism by which hypoxia diminishes the etoposide-induced apoptosis could be through an inhibition of the upregulation of *BAX *and *BAK1*, two pro-apoptotic genes. Erler et al [38] showed that hypoxia alone could decrease Bax (and Bid) expression and that this downregulation could participate to the cell sensitivity to etoposide. This is in accordance with our results. All together, these results revealed two candidate genes which could be responsible for the anti-apoptotic effect of hypoxia.

The effects reported by Erler et al [38] occur via HIF-1 dependent and independent pathways. Similarly, the changes in gene expression described in Table 1 suggest p53 dependent and independent mechanisms triggered by etoposide while hypoxia also exerts effects which may depend on HIF-1 but also on other transcription factors. We thus investigated in the second part of the work, the activity of 10 transcription factors. The findings, summarized in Table 3, suggest that both etoposide and hypoxia alone or in combination profoundly affected the activity of numerous transcription factors, and this differentially according to the factor. Of note is the profile of p53 activity: p53 was activated by etoposide, inhibited by hypoxia and displayed an intermediate activity when both treatments were applied together, indicating that hypoxia diminished the etoposide-induced p53 activation. On the other hand, hypoxia strongly activated HIF-1 and this activation was not affected by etoposide. Complex regulation also occurred for AP-1, probably involving different subunits and with a maximal activity in the presence of etoposide and hypoxia.

There is mounting evidence that HIF-1 mediates cell survival and apoptosis resistance both under hypoxic and normoxic conditions [39-41]. This is largely due to alterations in cellular energy metabolism. GLUT-1 expression regulation is one such example [41]. Here, we showed that HIF-1α silencing prevented the protective effect of hypoxia against etoposide-induced apoptosis, which is in line with these previous reports. Moreover, this sensitization was correlated with the loss of the effect of hypoxia on *BAK1 *expression i.e. hypoxia inhibits the etoposide-induced increase in *BAK1 *expression which is parallel to the inhibition of apoptosis while HIF-1α siRNA blocked both. All together, these results suggest that it is probably by regulating Bak expression that HIF-1 induces, at least in part, cell resistance. It has to be mentioned that we failed to observe such a sensitizing effect of HIF-1α siRNA in a previous work [29], probably because the effect of HIF-1α silencing previously achieved was not high enough.

Simple comparison was made by hand aimed to highline parallelism between variation in gene expression and transcription factor activation in order to determine which factor targets which gene. One example of such a correlation is the additive effect of hypoxia and etoposide on the overexpression of *MCL1 *and *CLK1 *with the maximal activity of AP-1 in these conditions. Similarly, expression pattern of *IGFBP4, BAX, BAK1, MDM2, CDKN1A *and *GPX1 *looks parallel to the profile of p53 activity. However, with regard to the complexity of the changes observed in gene expression, knowing that transcription factors could interact one with each other, e.g. HIF-1 and p53 [12,42] or HIF-1 and AP-1 [21], unbiaised cluster analyses were performed to evidence other unexpected correlations.

The results indicate that neither AP-1 activity, nor c-myc activity did correlate with the expression of genes detectable with the array. On the other hand, cluster 7 correlates HIF-1 activity but also CREB/ATF-2 activity, with the changes in the expression of *ING *and *MAP2K1 *but also of *MCL1, BNIP3 *and *JUN*, three known HIF-1 target genes. Expected findings were also obtained in cluster 8 where a correlation exists between p53 DNA binding activity, p53 transcriptional activity and the expression of p53 target genes e.g. *CDKN1A, GPX1 *and *PCNA*. However, other p53 target genes (*BAX, MDM2, GADD45A*) are clustered in cluster 9 together with the profile of NF-kB activity and the expression profile of NF-kB target genes (*BIRC3 *and *BIRC2*). Cluster 1 correlates the expression profiles of genes whose function in apoptosis is less clear and does not contain any transcription factor activity profile. Further investigation will be needed to unravel the identity of the exact factor(s) responsible for the overexpression of these genes in the different conditions.

Our results indicated that in our experimental conditions, (1 % O_2_), hypoxia does not lead to NF-kB activation in HepG2 cells. There are reports in the literature reporting NF-kB activation under hypoxia in different cell types: macrophages [43], neutrophils [44], endothelial cells [45] as well as cancer cells [46]. The exact mechanism responsible for NF-kB activation under hypoxia is not clear but it may involve ROS [47]. However, there are also other reports that show that NF-kB is activated during the reoxygenation phase following a previous hypoxia incubation period and in these cases, ROS are clearly involved in NF-kB activation BUT there is NO activation of NF-kB during the hypoxia phase [48-50]. In conclusion, according to the experimental conditions and probably the cell types, hypoxia does or does not activate NF-kB but the exact mechanisms for this fine tuning are not yet known.

Finally, in order to delineate putative pathways involved in the anti-apoptotic effect of hypoxia, "phenotypic" profiles for caspase activity and DNA fragmentation were added for the cluster analysis. This new analysis generated different clusters than the previous one. DNA fragmentation profile correlates with the expression pattern of three p53 target genes (cluster 6), one of them being involved in DNA repair (*GADD45A*). On the other hand, caspase activity profile was clustered with p53 DNA binding activity and with the expression pattern of other p53 target genes among which is *BAX*, and *BAK1*. The reason why p53 transcriptional activity profile is not clustered in the same group is not known. It is intriguing to remark that up- and down-regulation of *BIRC2 *and *BIRC3*, which are anti-apoptotic genes, also correlates with caspase 3 activity profile, hence with the apoptotic state of the cells. Similarly, HIF-1 activity correlated with the overexpression pattern of *BNIP3*, which encodes a pro-apoptotic protein but also of *MCL1*, which encodes an anti-apoptotic protein. However, HIF-1 activity did not correlate with any "phenotypic" profile. These results suggest that it is probably a finely regulated balance of pro- and anti-apoptotic proteins that eventually tilts the cell fate towards life or death. It must also be noted that the changes in mRNA expression induced by etoposide and/or hypoxia may not always be reflected in the actual protein levels.

An interesting hypothesis is that HIF-1 could directly influence p53. We investigated the effect of HIF-1α siRNA on p53 mRNA level and on its activity by measuring the expression of some of its target genes (mRNA level for *BAX *and *GPX1*). It has to be mentioned that it is not possible to assess the effect of HIF-1α siRNA on p53 protein level (hence on its DNA binding activity): indeed the protein HIF-1α is only present under hypoxia conditions while p53 is markedly downregulated. Regarding p53 mRNA level, neither etoposide, nor hypoxia did influence this level. Moreover, HIF-1α siRNA had no effect either. Regarding p53 activity, the expression of its target genes (e.g. *GPX1 *and *BAX *mRNA) was increased in the presence of etoposide and decreased under hypoxia alone. This expression remained very low under hypoxia when etoposide was added. There was no effect of HIF-1α siRNA on p53 target genes in any of these conditions (see Table 4). From these results, we concluded that the effect of hypoxia on p53 is not mediated by HIF-1.

Our results indicate that under hypoxia, *BAK1 *may be downregulated through a HIF-dependent pathway while *BAX *is not. Since an inhibition of HIF-1 relieved the protection against the hypoxia-induced inhibition of etoposide-induced apoptosis, which occurred in parallel to a re-expression of *BAK1*, we hypothesis that HIF-1-dependent *BAK1 *expression may be involved in the protection brought by hypoxia. It does not exclude that hypoxia exerts its effect via other mechanisms. Indeed, a clear inhibition of p53 is observed under hypoxia, as well as of *BAX *expression, that both can also participate to the protection observed under hypoxia. These two effects are however HIF-1-independent, since that still occurred when HIF-1 was inhibited.

In conclusion, our data evidenced complex changes in the activity of numerous transcription factors and in the expression of various pro- and anti-apoptotic genes induced by etoposide and/or hypoxia. However, a system biology approach helped to define putative pathways that may be responsible for the anti-apoptotic effect of hypoxia, one of them being the inhibition of p53 activation and hence of the expression of some of its targets genes. *BAX *and more probably *BAK1 *are interesting candidates which can transduce the effect of hypoxia on HIF-1 and p53 activity and its actual effect on the induction of apoptosis. Additional studies are now needed to evaluate the exact implication of these proteins.

## Materials and methods

### Cell culture and hypoxia incubation

Human hepatoma cells HepG2 were maintained in culture in 75-cm2 polystyrene flasks (Costar) with 15 ml of Dulbecco's modified Eagle's medium liquid (DMEM) containing 200 U/ml penicillin and 200 μg/ml streptomycin (Biowhittaker Europe) and 10% of foetal calf serum and incubated under an atmosphere containing 5% CO_2_.

For hypoxia experiments (1 % O_2_), cells were incubated in serum-free CO_2_-independent medium (Invitrogen) supplemented with 1 mM L-glutamine (Sigma) with or without etoposide (Sigma) at 50 μM. Normoxic control cells were incubated in the same conditions but in normal atmosphere (20 % O_2_).

### Clonogenic assay

Cells were seeded at 3,000 cells per well (6 well plates) 24 hours before the incubation. They were then incubated for 16 hours in serum-free CO_2_-independent medium in the different experimental conditions and finally 7 days in complete medium. Cells were stained with crystal violet.

### DNA fragmentation

The measurement of cytoplasmic histone-associated DNA fragments (mono- and oligonucleosomes) after induction of cell death was performed with the « cell death detection ELISA » (Roche Molecular Biochemicals).

### Caspase activity

The measurement of total caspase activity was performed with the "Homogeneous Caspases Assay" kit (Roche Molecular Biochemicals). The assay for caspase 3 activity was performed according to Cosse et al [51].

### LDH release

LDH release was measured with the « cytotoxicity detection kit » from Roche Molecular Biochemical according to the manufacturer's protocol. The culture media from incubated cells were removed and centrifuged to pellet the cell fragments and apoptotic bodies. In order to lyse the cells, Triton X100 (Merck) at 10 % in PBS was added on this pellet as well as on the cells remaining in the wells. The percentage LDH release was calculated as follows:

LDH activity in medium (1) + LDH activity of cell fragments (2)/(1) + (2) + LDH activity of cells remaining in the wells.

### Western blotting

HepG2 cells, seeded in 25 cm^2 ^flasks, were scrapped in 200 μl of lysis buffer (Tris 40 mM pH 7.5, KCl 150 mM, EDTA 1 mM, triton X-100 1%) containing a protease inhibitor mixture (« Complete » from Roche Molecular Biochemicals, 1 tablet in 2 ml H_2_O, added at a 1: 25 dilution) and phosphatase inhibitors (NaVO_3 _25 mM, PNPP 250 mM, α-glycerophosphate 250 mM and NaF 125 mM, at a 1: 25 dilution). Western blot analysis was performed as described in [29] using mouse anti-PARP-1 monoclonal antibody (#556493 from Pharmingen) or mouse anti-HIF-1α (#610958 from Beckton Dickinson) monoclonal antibody as the primary antibody.

### Immunofluorescence staining and confocal microscopy

Immunofluorescence staining was performed as described in [29] using rabbit anti-active caspase 3 (#67481 Promega) as the primary antibody.

### Nuclear protein extraction

Nuclear protein extractions in high salt buffer were prepared as previously described [52]. Briefly, HepG2 cells seeded in 75 cm^2 ^flasks (Corning) were incubated with or without etoposide under normoxic or hypoxic conditions for 16 hours. At the end of the incubation, cells were rinced with PBS containing 1 mM Na_2_MoO_4 _and 5 mM NaF and incubated on ice for 3 minutes with 10 ml cold Hypotonic Buffer (HB, 20 mM HEPES, 5 mM NaF, 1 mM Na_2_MoO_4_, 0.1 mM EDTA) and harvested in 500 μl HB containing 0.2% NP-40 (Sigma), a protease inhibitor cocktail (Roche) and phosphatase inhibitors (1 mM Na_3_VO_4_, 5 mM NaF, 10 mM p-nitrophenylphosphate, 10 mM β-glycerophosphate). Cell lysates were centrifuged 30 seconds at 13000 rpm and sedimented nuclei were resuspended in 50 μl HB containing 20% glycerol and protease/phosphatase inhibitors. Extraction was performed for 30 minutes at 4°C by the addition of 100 μl HB containing 20% glycerol, 0.8 M NaCl and protease/phosphatase inhibitors.

### TF Chip MAPK microarray

TF Chip MAPK microarrays (Eppendorf) were used to analyze the activation state of eight transcription factors, namely AP1 (c-Jun), ATF-2, c-Myc, Elk-1, MEF2, NFATc1, STAT1 and p53. Each array contains triplicate spots of double-stranded DNA molecules containing the binding sequence for the corresponding transcription factor, and positive and negative controls which are used to normalize the data. Arrays were contacted with 30 μg nuclear extracts, and detection was performed in fluorescence, according to the manufacturer's protocol. Scanning was performed using a ScanArray scanner (Perkin Elmer).

### DNA-binding assay

DNA-binding assays using TransAM ELISA kit (Active Motif) for detecting transcription factor DNA binding activity was performed according to the manufacturer's recommendations. Briefly, 10 μg of nuclear proteins were incubated for 2 hours in a 96-well plate coated with a double-stranded oligonucleotide containing the consensus sequence recognized by the transcription factor to be assayed. The transcription factor bound to DNA was detected using a specific primary antibody. Colorimetric reaction was then performed with a HRP-conjugated anti-rabbit IgG antibody and absorbance was measured at 450 nm in a spectrophotometer.

### HIF-1a siRNA transfection

Knockdown of HIF-1α expression was achieved using siGENOME SMARTpool human HIF1A from Dharmacon. siCONTROL non-targeting siRNA#1 was used to control for non-specific effects. Cells were transfected 24 hours under standard culture conditions with 50 nM siRNA using the DharmaFECT 1 (Dharmacon) transfection reagent according to the manufacturer's instructions. Cells were then incubated under hypoxia for 16 hours.

### Transient transfection and luciferase assay

HepG2 transfections were performed in 24-well plates (50,000 cells per well) with SuperFect reagent (Qiagen). 1846 ng of the reporter plasmid containing binding sites for the transcription factor to be assayed upstream of the firefly luciferase gene were co-transfected with 1154 ng of normalization vector (pCMVb vector coding for the β-galactosidase, Promega) in DMEM without serum for 8 hours. Reporter plasmids were pGL3-SV40/6HRE vector containing 6 HRE binding sites upstream of the firefly luciferase gene [53], pAP1-Luc (Stratagene), pNF-kB-Luc (Stratagene), pCRE-Luc (Stratagene), pG13-Luc containing 13 copies of a p53-responsive promoter driving the expression of the luciferase gene [54] and pGL2-M4-luciferase containing 4 c-myc binding sites upstream of the firefly luciferase gene [55]. Cells were then directly incubated under hypoxia for 16 hours. After hypoxia incubation, β-galactosidase was assayed in parallel to the firefly luciferase activity, assayed in a luminometer using the Luciferase Reporter Assay System (Promega). Results are expressed as means of the ratio between the firefly luciferase activity and the β-galactosidase activity.

### Gene expression analysis on DNA microarray

We used a low-density DNA array allowing the gene expression analysis for 123 genes related to apoptosis (DualChip^® ^human apoptosis, Eppendorf). Results using these reliable and validated arrays developed by Eppendorf were reported elsewhere [56-58]. The method is based on a system with two identical arrays on a glass slide and three identical sub-arrays (triplicate spots) per array. HepG2 cells cultured in 75 cm^2 ^flasks (Corning) were incubated for 16 hours with or without etoposide under normoxic and hypoxic conditions. At the end of the incubation, total RNA was extracted with the Total RNAgents extraction kit (Promega), quality was checked with a bioanalyzer (Agilent Technologies) and 20 μg were used for retrotranscription in the presence of biotin-11-dCTP (Perkin-Elmer) and Superscript II Reverse Transcriptase (InVitrogen), as described previously [56]. Hybridizations on the arrays were carried out as described by the manufacturer and reported previously [56]. Detection was performed with a cyanin 3-conjugated IgG anti-biotin (Jackson Immuno Research Laboratories). Fluorescence of hybridized arrays was scanned using a Packard ScanArray (Perkin-Elmer) at a resolution of 10 μm.

### Real time RT-PCR

After the incubation, total RNA was extracted using the Total RNAgent extraction kit (Promega). mRNA contained in 5 μg total RNA was reverse transcribed using SuperScript II Reverse Transcriptase (InVitrogen) according to the manufacturer's instructions. Sequences of primers are available on request. Amplification reaction assays contained 1× SYBR Green PCR Mastermix (Applied Biosystem) and primers (Eurogentec) at the optimal concentrations and amplification was performed using an ABI PRISM 7000 SDS thermal cycler (Applied Biosystem). *α-tubulin *was used as the reference gene for normalization and mRNA expression level was quantified using the threshold cycle method.

### Cluster analysis

For cluster analysis, we used EPCLUST, a web-based clustering analysis and visualisation tool [59]. Log_10_-converted expression or transcription factor activity data were subjected to K-Means clustering in order to generate clusters of similar variation profiles. The distance computed between two profiles corresponds to the Manhattan distance.

## Competing interests

The author(s) declare that they have no competing interests.

## Authors' contributions

JPC, AS and MC carried out all the experiments, NN carried the immunofluorescence studies, VM developed the TFChip MAPK assay, FdL developed the DualChip human apoptosis, MR and JR participated in the design of the study, CM conceived the study, participated in its design and coordination and helped to draft the manuscript. All authors read and approved the final manuscript.

## Supplementary Material

Additional file 1 Effect of hypoxia and/or etoposide on protein abundance and subcellular localization of different transcription factors. Cells were incubated under normoxic (N) or hypoxic (H) conditions with or without etoposide (e) at 50 μM for 5 hours. After the incubation, cells were fixed, permeabilized and stained for HIF-1α, p53, phospho-serine 15 p53, c-jun and phospho-serine 63 c-jun, using specific antibodies (green). Nuclei were detected with To-Pro-3 (blue). Observation was performed using a confocal microscope with the photomultiplier constant. Please refer to supplementary data (fig. 3.S) for results obtained for c-fos, c-myc, phospho-elk, phospho-ATF-2, phospho-CREB, STAT-1α, p65, p50, c-rel and MEF-2. Primary antibodies were as follows: mouse anti-p53 (#05-224 Upstate), mouse anti-phospho serine 15-p53 (#92865 Cell Signaling), rabbit anti-c-jun (SC-1694 Santa Cruz), rabbit anti-phospho serine 63-c-jun (#92615 Cell Signaling), rabbit anti-c-fos (SC-052 Santa Cruz), mouse anti-phospho serine 83-elk-1 (SC-8406 Santa-Cruz), mouse anti-phospho threonine 71-ATF2 (SC-8398 Santa Cruz), rabbit anti-phospho serine 133-CREB (#06-519 Upstate), rabbit anti-MEF-2 (SC-10794 Santa Cruz), mouse anti-c-myc (SC-42 Santa Cruz), rabbit anti-STAT-1α (SC-345 Santa Cruz), mouse anti-c-rel (SC-6955 Santa Cruz), rabbit anti-p50 (SC-7178 Santa Cruz), rabbit anti-p65 (SC-372 Santa Cruz), mouse anti-HIF-1α (#610958 BD Biosciences). Click here for file

Additional file 2 Effect of hypoxia and/or etoposide on DNA binding activity of 8 transcription factors measured with the TransAM assays. Cells were incubated under normoxic (N) or hypoxic (H) conditions with or without etoposide (e) at 50 μM for 16 hours. After the incubation, nuclear extracts were obtained from three independent experiments and hybridized in the ELISA well containing specific DNA probes. Detection was performed using specific antibodies. Results are expressed in absorbance (means ± 1 SD, n = 3). *, **, ***: p < 0.05, p < 0.01, p < 0.001 vs. normoxia ; §§, §§§: p < 0.01, p < 0.001 vs. normoxia+etoposide. Click here for file
