# Supplementary figures and images for: Hypoxia induces protection against etoposide-induced apoptosis: molecular profiling of changes in gene expression and transcription factor activity
Source: Mol Cancer. 2008 Mar 26;7:27. doi: 10.1186/1476-4598-7-27 (PMC2330149; doi:10.1186/1476-4598-7-27)

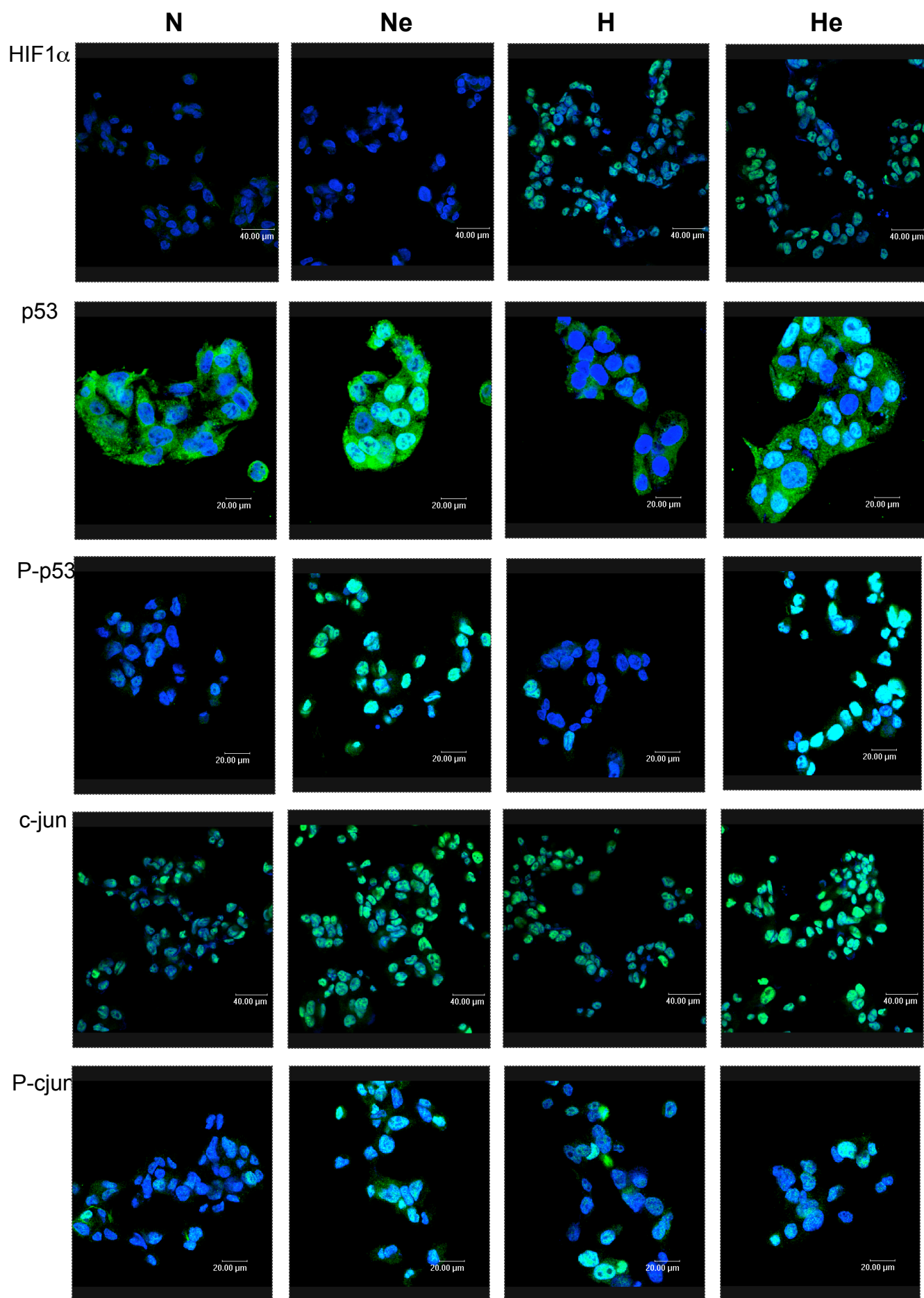

figure 1S

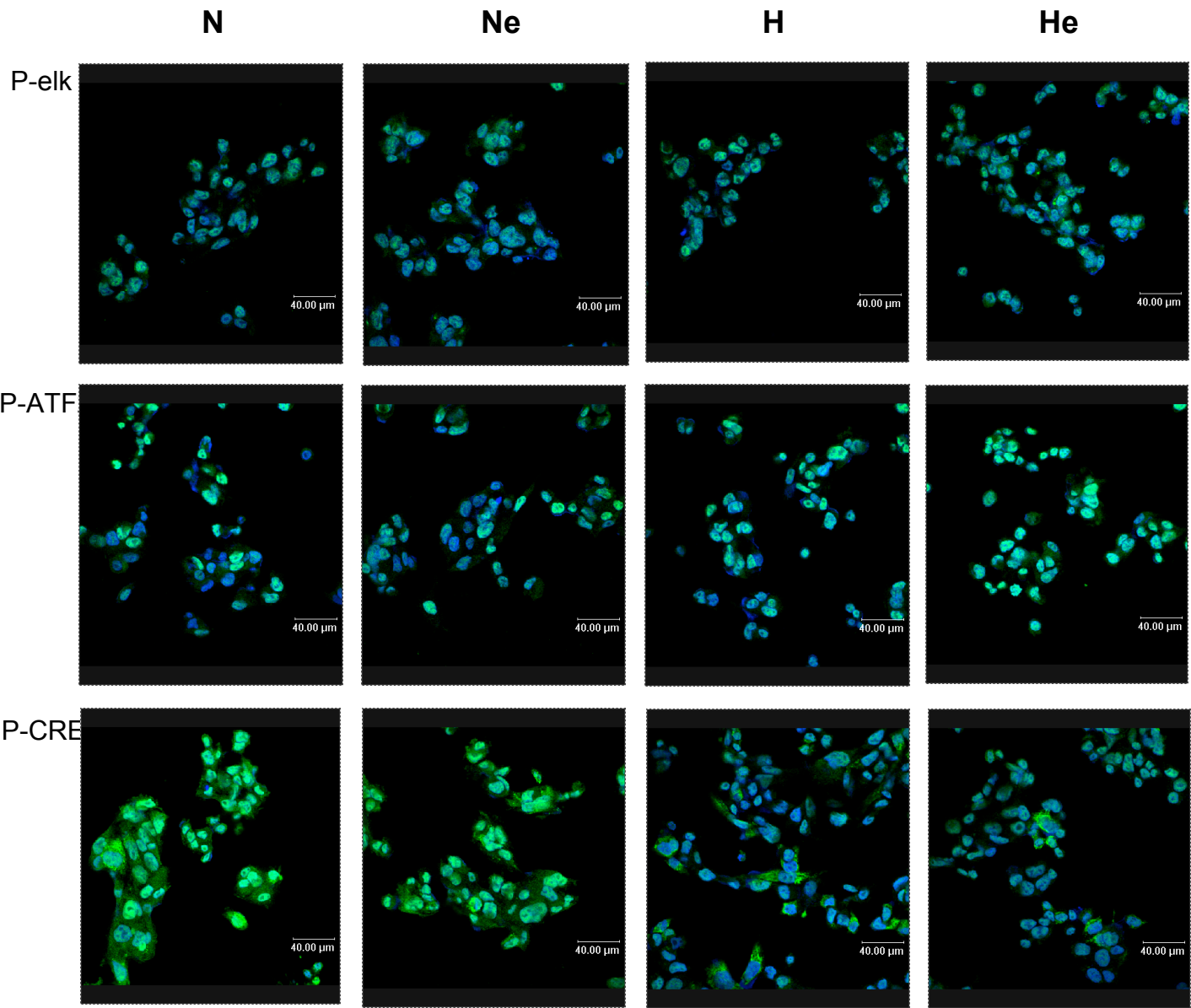

figure 1S

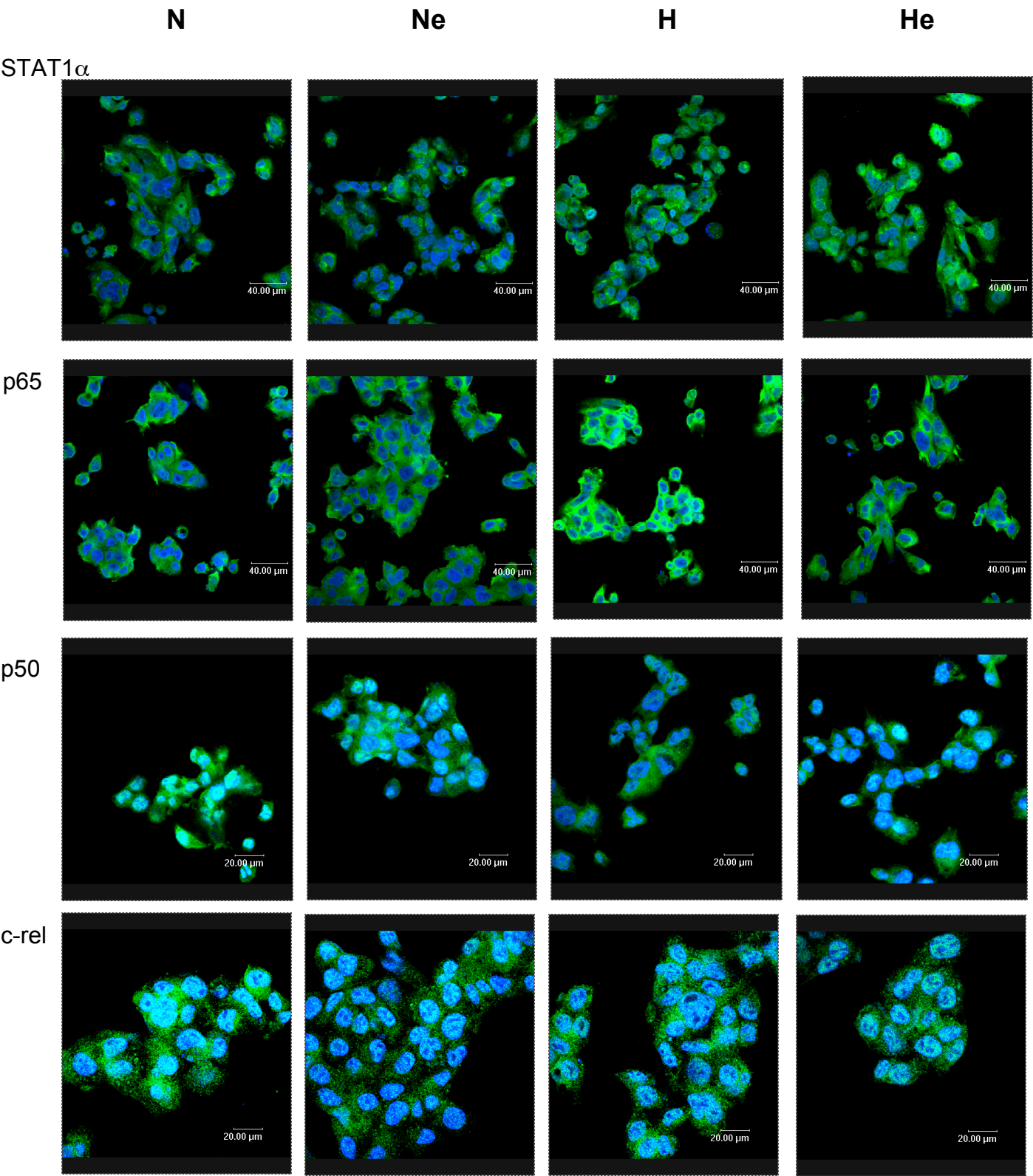

figure 1S

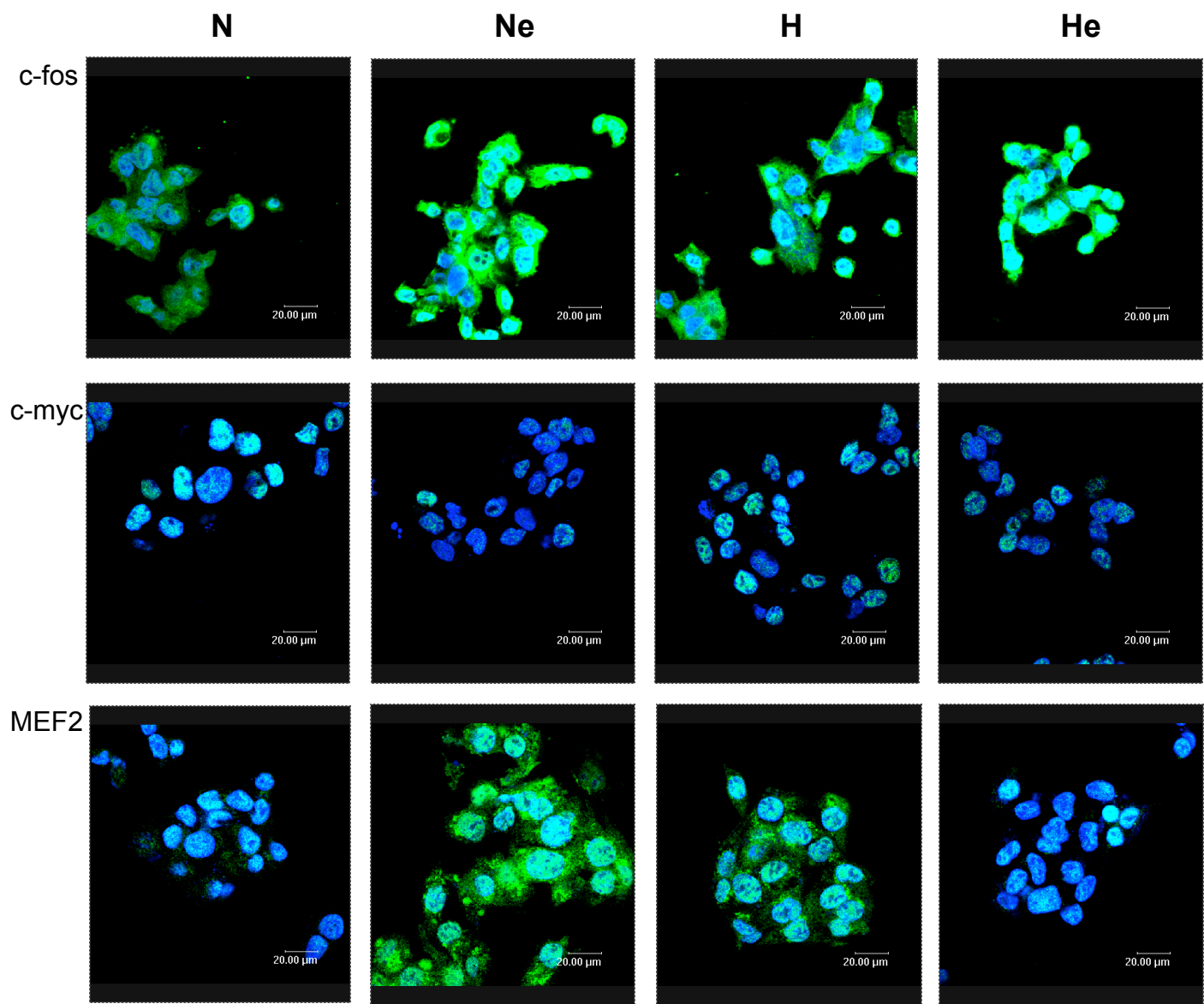

Supplement: Additional file 1 — Effect of hypoxia and/or etoposide on protein abundance and subcellular localization of different transcription factors. Cells were incubated under normoxic (N) or hypoxic (H) conditions with or without etoposide (e) at 50 μM for 5 hours. After the incubation, cells were fixed, permeabilized and stained for HIF-1α, p53, phospho-serine 15 p53, c-jun and phospho-serine 63 c-jun, using specific antibodies (green). Nuclei were detected with To-Pro-3 (blue). Observation was performed using a confocal microscope with the photomultiplier constant. Please refer to supplementary data (fig. 3.S) for results obtained for c-fos, c-myc, phospho-elk, phospho-ATF-2, phospho-CREB, STAT-1α, p65, p50, c-rel and MEF-2. Primary antibodies were as follows: mouse anti-p53 (#05-224 Upstate), mouse anti-phospho serine 15-p53 (#92865 Cell Signaling), rabbit anti-c-jun (SC-1694 Santa Cruz), rabbit anti-phospho serine 63-c-jun (#92615 Cell Signaling), rabbit anti-c-fos (SC-052 Santa Cruz), mouse anti-phospho serine 83-elk-1 (SC-8406 Santa-Cruz), mouse anti-phospho threonine 71-ATF2 (SC-8398 Santa Cruz), rabbit anti-phospho serine 133-CREB (#06-519 Upstate), rabbit anti-MEF-2 (SC-10794 Santa Cruz), mouse anti-c-myc (SC-42 Santa Cruz), rabbit anti-STAT-1α (SC-345 Santa Cruz), mouse anti-c-rel (SC-6955 Santa Cruz), rabbit anti-p50 (SC-7178 Santa Cruz), rabbit anti-p65 (SC-372 Santa Cruz), mouse anti-HIF-1α (#610958 BD Biosciences). [file 1476-4598-7-27-S1.pdf]

**p53**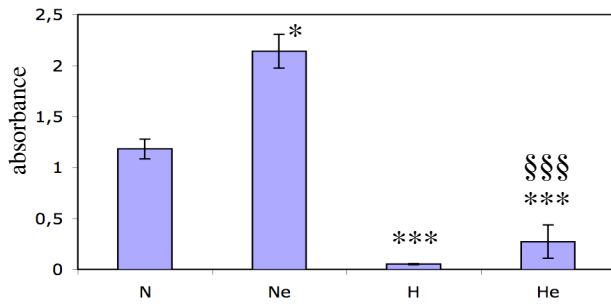**phospho-Elk-1**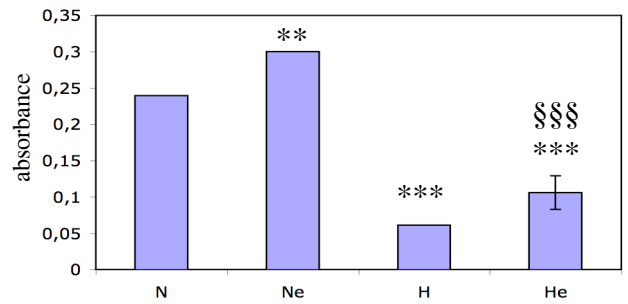**AP-1 (c-jun)**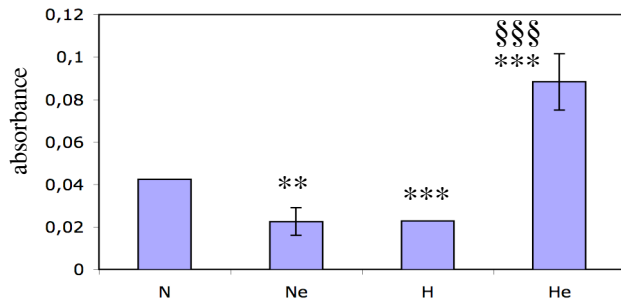**AP-1 (c-fos)**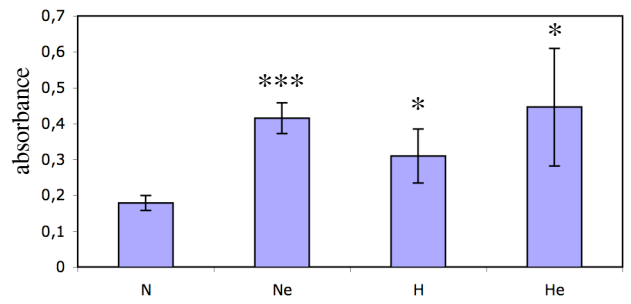**NF-κB (p65)**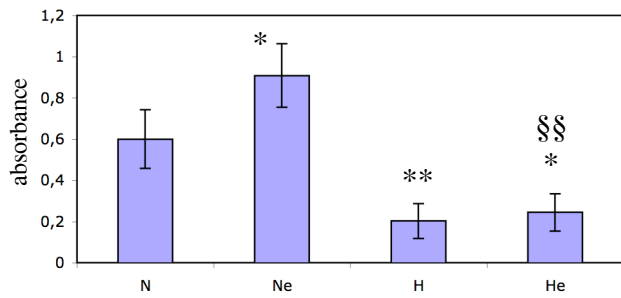**phospho-CREB**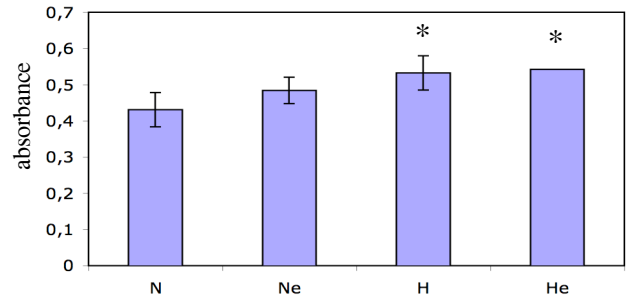**c-myc**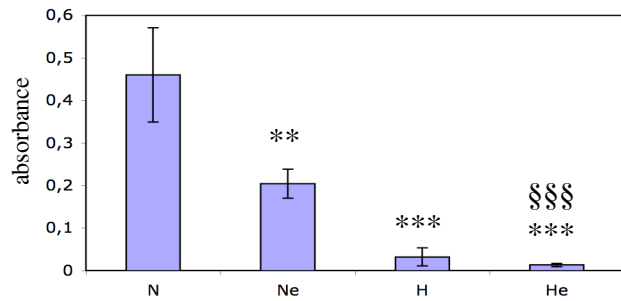**HIF-1**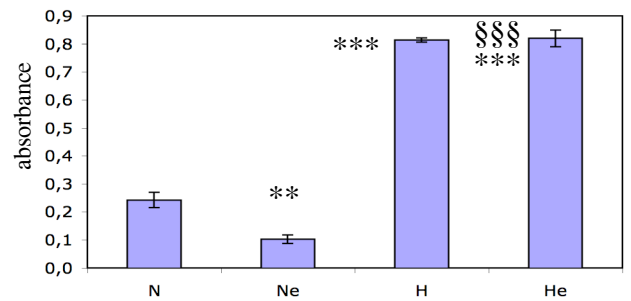

Supplement: Additional file 2 — Effect of hypoxia and/or etoposide on DNA binding activity of 8 transcription factors measured with the TransAM assays. Cells were incubated under normoxic (N) or hypoxic (H) conditions with or without etoposide (e) at 50 μM for 16 hours. After the incubation, nuclear extracts were obtained from three independent experiments and hybridized in the ELISA well containing specific DNA probes. Detection was performed using specific antibodies. Results are expressed in absorbance (means ± 1 SD, n = 3). *, **, ***: p < 0.05, p < 0.01, p < 0.001 vs. normoxia ; §§, §§§: p < 0.01, p < 0.001 vs. normoxia+etoposide. [file 1476-4598-7-27-S2.pdf]
